# Comparison of Bone Mineral Density and Treatment Initiation Among Treatment-Naive Post-menopausal Women With and Without Distal Radius Fracture: A Case-Control Study From Southern Europe

**DOI:** 10.7759/cureus.82414

**Published:** 2025-04-17

**Authors:** Vasileios Giannatos, Irini Tatani, Konstantinos Stathopoulos, Antonios Galanos, Panagiotis Antzoulas, Andreas Panagopoulos

**Affiliations:** 1 Orthopaedics, School of Medicine, University of Patras, Patras, GRC; 2 Orthopaedics, Laboratory for Research of the Musculoskeletal System, National and Kapodistrian University of Athens, Athens, GRC; 3 Epidemiology and Public Health, Laboratory for Research of the Musculoskeletal System, KAT General Hospital, School of Medicine, National and Kapodistrian University of Athens, Athens, GRC

**Keywords:** distal radius, osteopenia, osteoporosis, post-menopausal, vitamin d

## Abstract

Objectives

The objective of this study is to compare bone mineral density (BMD), Fracture Risk Assessment Tool (FRAX) scores, comorbidities, risk factors, and biochemical blood markers among postmenopausal women with distal radius fractures and those without such fractures.

Materials and methods

Sixty-three postmenopausal women with distal radius fractures were compared to 64 postmenopausal women with no history of fractures. Primary objectives included comparing BMD at the femoral neck and lumbar spine, as well as FRAX scores between the two groups. Secondary goals involved assessing comorbidities, risk factors, and biochemical blood markers (Ca+2, albumin, parathormone (PTH), and vitamin D (VITD)) between the two groups.

Results

BMD and T-scores for the hip and lumbar spine were compared between groups, revealing no statistically significant differences. However, FRAX scores (major and hip) differed significantly in the fracture group (p = 0.005), aligning with expectations that a previous fracture elevates the FRAX risk for future fractures. Biochemical markers were similar between the two groups, except for vitamin D. Among risk factors and comorbidities, only multi-drug regimens and epileptic seizures were significantly higher in the fracture group.

Conclusion

Our study found similar BMD between the two groups. Nonetheless, a lower FRAX in the fracture group necessitates increased consideration for osteoporosis treatment. Multiple-drug regimens and lower vitamin D levels were also linked to the fracture group.

## Introduction

Osteoporosis is a systemic skeletal disorder characterized by low bone mineral density (BMD), micro-architectural deterioration of bone tissue, and consequent increase in fracture risk [1-3]. Osteoporosis is classified into two types: postmenopausal osteoporosis (type 1) occurs in women within 15-20 years after menopause, is related to or exacerbated by estrogen deficiency, and is usually presented with fractures in the distal radius and spine. Age-related osteoporosis (type 2) occurs in men and women over 75 years of age and may be more directly related to reduced osteoblastic activity, vitamin D deficiency, and calcium malabsorption; most common fractures occur in the hip, proximal humerus, pelvis, tibia, and ribs [4]. The morbidity and mortality of osteoporotic fractures in the elderly is a major healthcare concern, especially regarding hip fractures, as it has been estimated that they present up to 30% mortality within the first year in male patients [5-7]. Mortality rates of up to 20% have also been observed in osteoporotic vertebral fractures, mainly due to weight loss, frailty, and pulmonary dysfunction [8-11]. For this reason, numerous studies have been dedicated to the treatment and prevention of these fractures.

Distal radius fractures show a bimodal age distribution occurring more commonly in the pediatric population, during sporting activities, around the time of puberty when bone mineralization is low, and in the elderly population, mainly women, after a fall from a standing height [12-13]. In the elderly population, distal radius fractures are the second most common fracture after hip fractures, affecting their overall performance and daily living activities [12-13]. Over the age of 50, in both genders, distal radius fractures have been associated with higher rates of osteoporosis as measured by dual-energy X-ray absorptiometry (DEXA) and low vitamin D levels and are thus considered as a predisposing factor for early diagnosis of osteoporosis and prevention of future hip or spinal fractures [14-21]. The American Academy of Orthopaedic Surgeons (AAOS) proposed in 2009 the indications for surgical management of distal radius fractures, consisting of post-reduction radial shortening > 3 mm, dorsal tilt > 10°, and intra-articular displacement or step-off > 2 mm [22]. However, in patients older than 65 years old or in patients with limited functional demands, conservative treatment in a cast is the gold standard due to postoperative complication rates reaching 33% (vs. 14% for conservative treatment) and no obvious clinical superiority in this particular population, according even to the latest AAOS guidelines of 2020 [23-26]. While not much can be offered regarding fracture treatment in this population, with conservative and surgical options both presenting with their downsides, prevention of future fractures can have a large impact, being life-saving for these patients. Post-fracture investigation of osteoporosis and the prevention of secondary fractures has been a hot topic of discussion during the last years, with fracture liaison services intervening in the most developed countries [27-31].

In the literature, only a few case-control studies exist directly comparing the BMD among post-menopausal women with distal radius fractures and not [15]. In the present study, we conducted a retrospective observational case-control study comparing the prevalence of osteoporosis among women >50 years old with a distal radius fracture with those without a fracture from our center.

## Materials and methods

Our study is a retrospective observational case-control study conducted in our Metabolic Bone Diseases Outpatient Clinic from January 2018 to June 2021. Women above 50 years old who presented to our emergency department with distal radius fractures were referred to our osteoporosis outpatient clinic for a secondary evaluation. The control group consisted of women over 50 years old with no fracture history who had scheduled appointments at our osteoporosis clinic. All patients were informed about the study protocol and provided written informed consent for publication. Before the initiation of the study, the protocol was approved by the Universital Hospital of Patras’ ethics committee, and Institutional Review Board approval was obtained (University Hospital of Patras, approval number 684/12.10.18-56/21.11.2018). Inclusion criteria were as follows: 1) female gender, 2) age >50 years, and 3) distal radius fracture (for the fracture group). Exclusion criteria were as follows: 1) secondary osteoporosis and 2) high-energy fracture (motor vehicle accident, fall from height >2 m).

The primary goal was to compare osteoporosis status according to BMD values at the lumbar spine and femoral neck, as well as the Fracture Risk Assessment Tool (FRAX).

Secondary goals were to compare other clinical risk factors and comorbidities between the two groups, namely, thyroid disease, dyslipidemia, hypertension, gastrointestinal disorders, arrhythmia, emotional disorders and epileptic seizures, antidepressants, thyroxine, smoking, parental history of hip fracture, cortisone intake, diabetes, chemotherapy, early menopause, history of fracture, family history of fracture, chronic obstructive pulmonary disorder (COPD), multiple drug regimen, rheumatoid arthritis, protein pump inhibitors (PPIs), and >3 falls/year, furosemide. A multiple-drug regimen is defined as the systematic consumption of five or more different medications. Biochemical blood tests were also analyzed, including serum calcium, albumin (corrected calcium to albumin), 25-hydroxyvitamin (OH) vitamin D, and parathormone (PTH) levels.

Patients were assigned to two groups. Group 1 was the fracture group, which included women older than 50 years old with a recent history of distal radius fracture (within the last three years). Group 2 included women older than 50 years old with no recent fracture history who had been appointed in the outpatient osteoporosis clinic. No matching was performed between the two groups. The total BMD values were recorded for both the lumbar spine (L1-L4) and femoral neck using the same DEXA machine operated by the same technician (P.G.). The FRAX score was also calculated.

At the same time, blood samples were collected and analyzed for calcium, albumin, PTH, and vitamin D. A thorough medical history was collected by a consultant orthopedic surgeon (I.T.), including age, weight, height, comorbidities, medications, height loss, and other risk factors as described above. Treatment of osteoporosis, when detected, was offered according to the international guidelines.

Statistical analysis

Data were expressed as mean ± standard deviation (SD) or median, IQR (in case of violation of normality) for continuous variables, and as frequencies and percentages for categorical variables. The Kolmogorov-Smirnov test was utilized for normality analysis of the parameters.

The comparisons of the continuous variables between groups were analyzed using the independent samples t-test or the Mann-Whitney U test in case of violation of normality. The comparisons of the categorical variables between groups were analyzed using the χ² test of Fisher’s exact test.

All tests were two-sided, and statistical significance was set at p < 0.05. All analyses were carried out using the statistical package SPSS (IBM SPSS Statistics for Windows, IBM Corp., Version 21, Armonk, NY).

## Results

This article was previously posted to the Research Square preprint server on February 7, 2025 [32]. The total number of patients initially considered was 388. After applying the inclusion and exclusion criteria, 127 patients were enrolled in the study and assigned to two groups. Group A included 63 patients with distal radius fractures, and group B had 64 patients without any fracture history. Table 1 shows the baseline demographic characteristics of the two groups. The only statistically significant demographic parameter was age (p = 0.008), with a mean age in group A of 64.3 years vs. 60 years in the non-fracture group. The BMD and T-scores for both hip and lumbar spine were compared between groups, but no statistically significant difference was found (Table 1).

**Table 1:** Comparison of parametrical variables BMD: bone marrow density, SD: standard deviation

	Non-fracture		Distal radius fracture		p-value
	Mean	SD	Mean	SD	
Age	60.06	7.29	64.33	10.22	0.008
Weight	68.79	11.08	70.38	15.87	0.516
Height	1.58	0.06	1.57	0.08	0.807
ΒΜΙ	27.81	4.81	28.41	5.81	0.525
T-score lumbar spine	-1.69	0.83	-1.60	1.08	0.603
BMD lumbar spine	0.87	0.09	0.88	0.14	0.466
T-score femoral neck	-1.31	0.67	-1.41	0.95	0.502
BMD femoral neck	0.71	0.07	0.70	0.12	0.481

**Figure 1:**
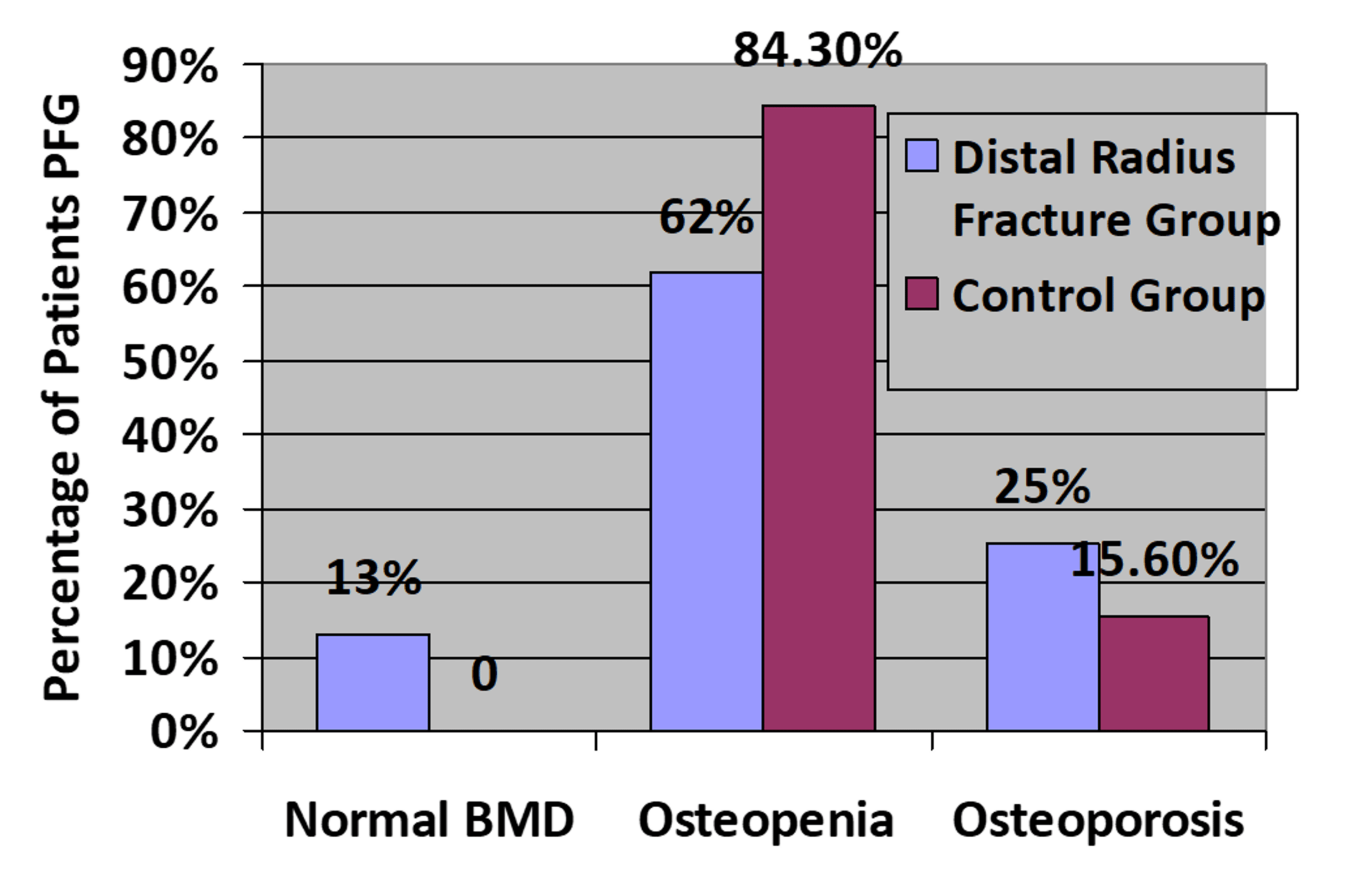
Results of osteoporotic status BMD: bone marrow density, PFG: per fracture group

In the fracture group, 16 patients were diagnosed with osteoporosis, and 39 had osteopenia. In the no-fracture group, 10 patients were found to have osteoporosis, and 54 patients were diagnosed with osteopenia, as depicted in Figure 1.

The metabolic and biochemical values (PTH, albumin, and corrected calcium) were found to be similar between the two groups, but vitamin D showed a significant difference (p = 0.044) with mean values of 25.82 and 20.82 in the non-fracture and fracture groups, respectively (Table 2).

**Table 2:** Comparison of non-parametrical variables 25-OH: hydroxyvitamin, ALB: albumin, Ca: calcium, FRAX: Fracture Risk Assessment Tool, IQR: inter-quartile range, PTH: parathormone, VTID: vitamin D

	Non-fracture		Distal radius fracture		p-value
	Median	IQR	Median	IQR	
25-OH VITD	22.94	16.42	19.04	12.00	0.044
PTH	48.95	20.08	49.20	19.40	0.687
ALB	4.00	0.30	4.20	0.40	0.180
Corrected serum Ca	9.34	0.46	9.20	0.42	0.102
FRAX major	5.40	4.72	9.40	7.80	<0.005
FRAX hip	1.05	1.53	1.80	3.70	<0.005

The FRAX scores (major and hip) were significantly different in the fracture group (p = 0.005), as was expected, because a previous fracture increases the FRAX risk for another fracture in the future (Table 2).

Comorbidities were also analyzed, but no differences were found between the two groups for thyroid disease, dyslipidemia, hypertension, emotional disorders, gastrointestinal disorders, arrhythmia, and epileptic seizures (Table 3). It is of interest, however, that epileptic seizures showed a prevalence of 0% and 6.3% in the non-fracture and fracture groups, respectively (p = 0.058). Other proven risk factors were also compared, and only multi-drug regimens were found to be significantly higher in the fracture group (p = 0.001, 0% and 14.3%, respectively). Antidepressants, thyroxine, smoking, family history of osteoporosis, diabetes, chemotherapy, cortisone, early menopause, history of fracture, history of fracture of mother, COPD, rheumatoid arthritis, >3 falls/year, furosemide, and PPIs showed similar prevalence between the two groups.

**Table 3:** Comparison of comorbidities COPD: chronic obstructive pulmonary disease, DEXA: dual-energy X-ray absorptiometry

	Non-fracture	Distal radius fracture	p-value
	Ν(%)	Ν(%)	
Thyroid disease	23.4%	23.8%	1.000
Dyslipidemia	39.1%	27.0%	0.187
Hypertension	32.8%	34.9%	0.853
Emotional disorders	14.1%	14.3%	1.000
Gastrointestinal disorders	4.7%	7.9%	0.492
Arrhythmia	6.3%	6.3%	1.000
Epileptic seizures	0%	6.3%	0.058
Antidepressants	9.4%	17.5%	0.203
Thyroxine	18.8%	12.7%	0.466
Smoking	43.8%	34.9%	0.365
Osteoporosis (mother)	23.4%	17.5%	0.510
Diabetes	4.7%	11.1%	0.206
Cortisone	4.7%	3.2%	1.000
Chemotherapy	3.1%	0%	0.496
Early menopause	23.4%	17.5%	0.510
History of fracture	12.5%	7.9%	0.560
History of fracture (mother)	9.4%	7.8%	1.000
COPD	1.6%	3.2%	0.619
Multi-drug regimen	0%	14.3%	0.001
>3 falls/year	0%	3.2%	0.244
Rheumatoid arthritis	0%	3.2%	0.244
PPIs	3.1%	6.3%	0.440
Furosemide	1.6%	3.2%	0.619
Risk factors ≥ 1	65.6%	100%	<0.0005
Risk factors ≥ 2	26.6%	54%	<0.002
Height loss	40.6%	60.3%	0.033
Need for treatment	25%	50.8%	0.003
DEXA osteoporosis	15.6%	25.4%	0.193

**Figure 2:**
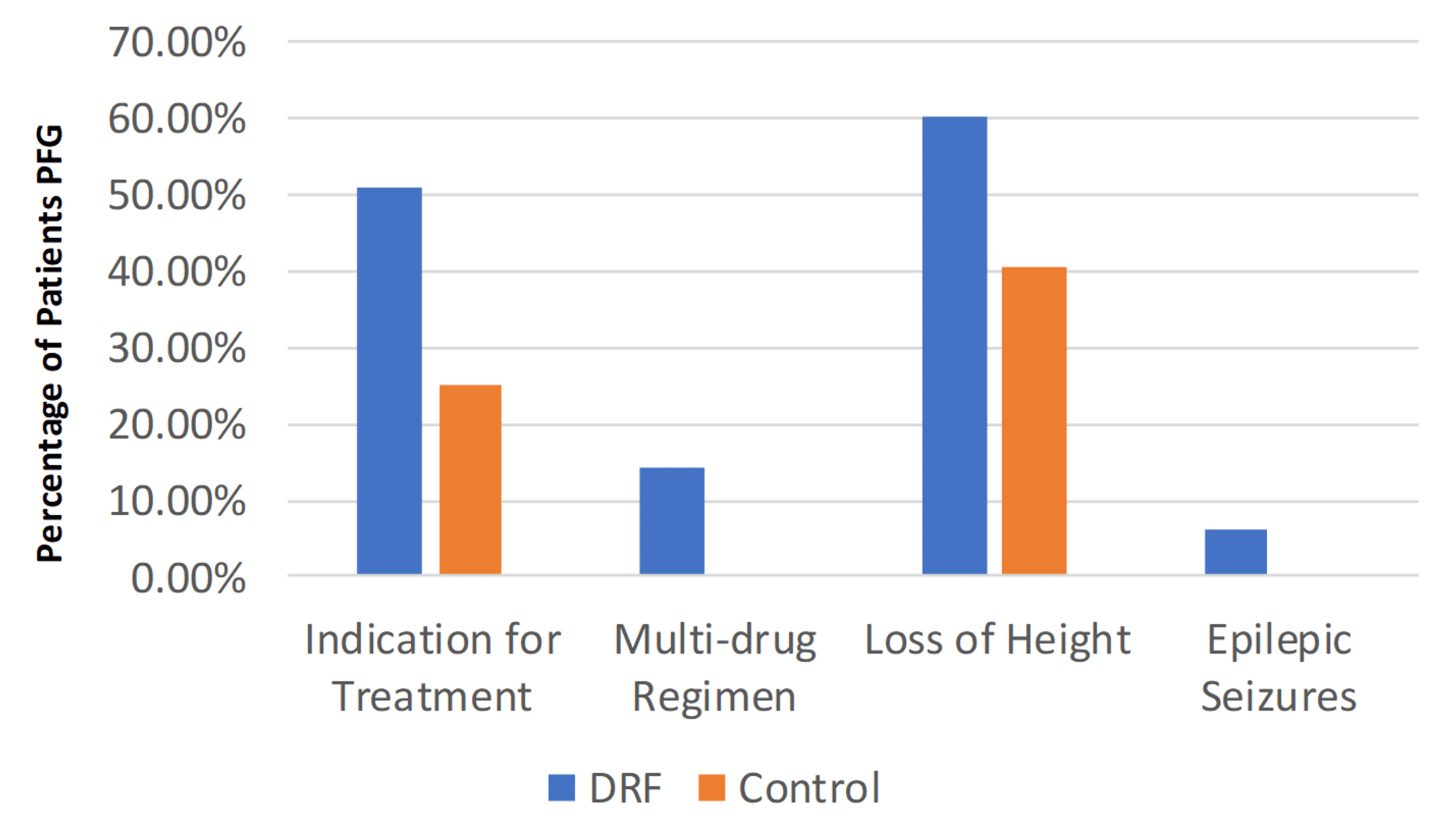
Results after statistical analysis DRF: distal radius fracture, PFG: per fracture group

Finally, the presence of ≥1 and ≥2 risk factors showed high p-values between the two groups; 100% of the fracture-group patients recorded at least one risk factor, and 54% at least two. On the contrary, 65.6% and 26.6% of the non-fracture group showed at least one or two risk factors, respectively. The loss of height also showed significance (p = 0.033), with 40.6% and 60.3% between the non-fracture and fracture patients. The need for antiosteoporotic treatment, according to the latest guidelines, was 25% and 50.8% for the control and fracture groups, respectively (p = 0.003) (Figure 2).

## Discussion

Osteoporotic distal radius fractures, particularly among women >50 years old, have been a topic of interest during the last decade, as they constitute the second most common fracture managed in the clinical setting [24,28-30]. A treatment plan is not the only parameter raising interest, as osteoporosis prevalence and morbidity rise, and a distal radius fracture in a middle-aged woman might be the first sign of the disease, requiring immediate treatment in that direction [30-31]. Other studies indicate that distal radius fractures might be the first signs of sarcopenia and frailty syndrome [33]. Even with normal BMD values, these patients remain at high risk for falls and subsequent fractures, and the interventions should follow a multidisciplinary approach to prevention, including vision problems, exercise, and environmental modifications [34].

Most similar studies concluded that distal radius fractures in women >50 years old are characterized by lower ultra-distal radius BMD [17,35-36]. However, less and conflicting evidence exists regarding BMD at the hip and lumbar spine and general osteoporotic status [17,19-20,37-38]. After a thorough literature review, we concluded that the majority of studies originate from northern Europe and found distal radius fracture in post-menopausal women to be correlated with higher osteoporosis prevalence [19-20,37-38]. Our study showed that women older than 50 years old with a distal radius fracture do not have lower BMD or T-scores than the non-fracture group of similar age, a difference that could be explained by the location of our study. Most studies showing a BMD difference among the groups were performed in Northern European countries, where it is known that osteoporosis presents a higher prevalence [16,39]. Our study, as well as the study from Barcelona, is the only study from the Mediterranean region that showed no difference in hip and lumbar spine BMD values among the two groups [17]. Nevertheless, there is a consensus that the risk for a subsequent hip fracture increases after a distal radius fracture in postmenopausal women [40], and this was confirmed by our study, as 50% of the fracture group presented with a need for anti-osteoporotic treatment after the FRAX score.

Among the different comorbidities and risk factors studied, only the multi-drug regimens showed a significant difference. It is not clear, however, whether this occurred due to the drugs’ side effects or due to the high comorbidities associated with multi-drug regimens. Loss of height was another statistically significant risk factor among fracture patients, and this indicates its potential prognostic value in this patient group, but extensive further clinical studies should be conducted in order to be incorporated into the treatment algorithm. Loss of height has been attributed to vertebral osteoporosis and fractures, along with kyphosis, and it is of interest whether it is correlated with such in this, younger than the usual vertebral osteoporotic group [41]. Nevertheless, it remains a standalone risk factor for another fracture, and high suspicion must be maintained when combined with distal radius fracture in post-menopausal women [42]. Epileptic seizures also showed a higher prevalence among the fracture group, although not statistically significant. Epileptic activity and osteoporosis share a complex and unclear relationship through various studies. Some studies suggest that the hypocalcemic effect of classic antiosteoporotic drugs might be severe enough to create seizures. However, our population was treatment-naive [43]. Other studies stress that antiepileptic agents can lower BMD, especially hepatic enzyme inducers like carbamazepine, phenobarbital, and phenytoin, while the frequency of seizures can increase during perimenopause, possibly increasing the fracture risk as well due to injury during epileptic episodes [44-46]. Finally, polypharmacy has raised interest during recent years among internists, as it has been linked with higher rates of morbidity and mortality among the elderly, but among osteoporosis specialists as well, as it has been linked with reduced rates of treatment compliance [47-48].

The reason that distal radius fractures in the elderly are so extensively studied in the literature is mainly due to the healthcare burden and morbidity of the accompanying osteoporotic hip and vertebral fractures. A Colles fracture after minor trauma is estimated to result in a subsequent hip fracture during the following years in 50% of the patients [26]. NICE guidelines have been suggested for secondary prevention of osteoporotic fractures, but most centers do not seem to widely implement these, as referral for further investigation should be initiated as early as in the emergency department [49-51]. Nevertheless, elderly patients with a low-energy distal radius fracture should be referred for a BMD measurement with DEXA, as there is a high prevalence of osteoporosis according to many studies, but not as high as to guarantee universal anti-osteoclastic treatment as in hip fractures [20,22]. Vitamin D and perhaps secondary osteoporosis laboratory tests should also be ordered, as Oyen et al. in 2011 showed a high prevalence of vitamin D inadequacy among these patients, although Rozental et al. did not confirm this finding, possibly due to regional differences [22,52]. Our study showed no differences in calcium levels between the two groups, and even calcium supplementation by Lamke et al. showed no difference [53]. Patient education and consultation by the treating surgeon are also proven methods of increased osteoporosis screening and should be implemented as much as possible [54].

Our study has a number of limitations. First of all, patients in the fracture group were found to be statistically significant older than the non-fracture group, although the difference was minor considering absolute numbers. Moreover, our group sizes were relatively small. However, this is one of the few studies in Greece from a special osteoporosis clinic with the collection of data performed by one researcher (I.T.), one technician, and one DEXA machine.

## Conclusions

Although distal radius fractures in middle-aged women have been associated with low BMD and have been identified as an osteoporosis precursor, our study did not verify this hypothesis. Regional differences seem to exist, showing that the distal radius fracture and its connection to osteoporosis are not fully understood. Nevertheless, this group of patients continues to present higher FRAX scores and, therefore, higher risk for future fractures, leading to higher treatment rates, but not high enough for universal treatment. Lower vitamin D among these patients should be considered as both a prevention target as well as a therapeutic target. Loss of height and multi-drug regimens are also met at a higher prevalence among the fracture group and could be utilized as part of prevention and early diagnosis strategies, as well as epileptic seizures.
